# False memory susceptibility is correlated with categorisation ability in humans

**DOI:** 10.12688/f1000research.4645.2

**Published:** 2014-10-16

**Authors:** Kathryn Hunt, Lars Chittka

**Affiliations:** 1 Biological and Experimental Psychology, School of Biological and Chemical Sciences, Queen Mary University of London, Mile End Road, London, E1 4NS, UK

## Abstract

Our memory is often surprisingly inaccurate, with errors ranging from misremembering minor details of events to generating illusory memories of entire episodes. The pervasiveness of such false memories generates a puzzle: in the face of selection pressure for accuracy of memory, how could such systematic failures have persisted over evolutionary time? It is possible that memory errors are an inevitable by-product of our adaptive memories and that semantic false memories are specifically connected to our ability to learn rules and concepts and to classify objects by category memberships. Here we test this possibility using a standard experimental false memory paradigm and inter-individual variation in verbal categorisation ability. Indeed it turns out that the error scores are significantly negatively correlated, with those individuals scoring fewer errors on the categorisation test being more susceptible to false memory intrusions in a free recall test. A similar trend, though not significant, was observed between individual categorisation ability and false memory susceptibility in a word recognition task. Our results therefore indicate that false memories, to some extent, might be a by-product of our ability to learn rules, categories and concepts.

## Introduction

When remembering the past, we typically feel that our memory allows retrieval of events as they really occurred. Yet a large body of work shows that memory is often surprisingly inaccurate, with errors ranging from misremembering minor details of events to generating illusory memories of entire episodes ( Loftus, 1997). False memory, the phenomenon of remembering something that actually never occurred, has become a widely studied topic since its origins in Binet’s (1900) *La Suggestibilité* and Bartlett’s (1932) *Remembering*. The pervasiveness of such false memories generates an evolutionary puzzle; in the face of selection pressure for accuracy of memory ( Dukas, 1999; Mery, 2013; Raine & Chittka, 2008), how could such systematic failures have persisted over evolutionary time? As with perceptual illusions, false memories might be inevitable by-products of otherwise adaptive cognitive processes. Here we explore whether individuals with a higher propensity to form false memories are better at other cognitive tasks, thus generating a trade-off by which certain cognitive capacities (in this case forming links between distinct memories, as in categorisation) cannot be achieved without the cost of memory inaccuracies.

A plethora of experimental paradigms exist for eliciting differing types of false memories in declarative memory, i.e. people’s conscious memory for facts ( Brainerd & Reyna, 2005). Episodic (and as such autobiographical) false memories are commonly elicited using the misinformation paradigm, in which information provided or questions asked after an event can bias memory ( Loftus, 2005). Conversely, semantic false memories can be elicited using the presentation of lists of semantically related words ( Deese, 1959; Roediger & McDermott, 1995). The so called Deese-Roediger-McDermott (DRM) paradigm has become widely used for exploring the malleability of memory. In this paradigm, participants begin by studying lists of words; for example a list may comprise the words *mad, fear, hate, rage, temper, fury, ire, wrath, happy, fight, hatred, mean, calm, emotion, enrage*. Each list is composed of the 15 strongest associates of one critically non-presented word, for example *anger* for the above list. Upon free recall of the lists or during a recognition test, the non-presented words are ‘remembered’ at high rates and with high levels of confidence. This high proportion of false memories is attributed to the strength of the associations between the words presented in the lists and the words falsely remembered ( Deese, 1959).

While such tests might be viewed as rather remote from real-life situations in which the accuracy of memory matters, including episodic memories ( DePrince *et al.*, 2004; Freyd & Gleaves, 1996), it has recently been proposed that different types of false memories may share, at least in part, the same underlying mechanisms ( Otgaar *et al.*, 2012; Zhu *et al.*, 2013). Otgaar *et al.*, (2012) showed that children who generate a rich false memory when subjected to a typical false memory implantation paradigm, such as being led to believe they once took a ride in a hot air balloon (which in fact never occurred), are also more susceptible to false memories in a DRM test than children who do not develop a rich implanted false memory. Thus the DRM paradigm, artificial though it may seem, can be a useful laboratory paradigm to test individual false memory susceptibility more generally (but see Ost *et al.*, 2013).

False memories, like other memory inaccuracies (such as forgetting) might be by-products of the otherwise adaptive nature of memory processes ( Howe *et al.*, 2013; Schacter, 1999; Schacter & Dodson, 2001; Schacter *et al.*, 2011). But what cognitive processes might facilitate the generation of false memories as a by-product? It is possible that our abilities for rule learning, association and categorisation might come at a cost when it comes to memorising isolated facts, events, or indeed words. Specifically with respect to the semantic false memories tested in the DRM paradigm, errors might be produced by the ability of individuals to group words together, placing them in categories based on rules for membership. It therefore seems plausible that the creation of these semantic false memories may be a by-product of our ability to group words into categories.

Categorising items is known to generate adaptive benefits such as the ability to learn information more quickly and to show greater efficiency during decision-making ( Merritt *et al.*, 2010), but McClelland (1995) argues that whilst such categorisation “is central to our ability to act intelligently” it however “gives rise to distortion as an inherent by-product” (p. 84). It is therefore possible that some memory errors are an inevitable fluke of a powerful, adaptive cognitive phenomenon, in the case of semantic false memories our ability to learn rules and concepts, and to classify novel objects by category memberships ( Carey, 2011; Chittka & Jensen, 2011). Indeed, categorisation is a strategy to economise on memory, since it allows recognising objects by a limited set of features that define the category, rather than memorising every single possible member of the category ( Avarguès-Weber *et al.*, 2011; Chittka & Niven, 2009; Srinivasan, 2006).

One possibility to explore the potential trade-off between categorisation ability and false memory susceptibility is to exploit variation between individuals, and to test whether superior performance on the one test comes with increased error rates on the other. Inter-individual variation is the raw material for evolution, and offers the possibility to quantify the fitness benefits of cognitive traits in natural settings ( Cole *et al.*, 2012; Raine & Chittka, 2008; Rowe & Healy, 2014; Thornton *et al.*, 2014) and to test potential trade-offs between one cognitive capacity and another ( Boogert *et al.*, 2011; Raine & Chittka, 2012). Here we investigate a potential correlation between an individual’s proneness to semantic type false memories and their categorisation ability. For this purpose we subjected participants to a DRM paradigm to assess their semantic false memory susceptibility and a test consisting of verbal reasoning questions to assess their ability to form categories. Our findings indicate that false memories, to some extent, might be a by-product of our ability to learn rules, categories and concepts.

## Methods

The general method for eliciting false memories was based on Roediger & McDermott (1995) and Stadler *et al.*, (1999). The protocol for the visual presentation of the wordlists was adapted from Peters *et al.*, (2008). The categorisation test was constructed from educational aids published by Coordination Group Publications Ltd ( Parsons, 2002a; Parsons, 2002b), Chukra Ltd, (2007) and Eleven Plus Exam Group (2010).

### Participants

Thirty-nine 2 ^nd^ year undergraduate students from the School of Biological & Chemical Sciences, Queen Mary University of London participated in the study. The participants were one full class undertaking a ‘statistics’ module and as such the experiment formed part of their learning, with a report writing task set from the results. Participant demographics were as follows: seven male, thirty-two female, aged nineteen to thirty years. Full ethics approval was obtained from Queen Mary University of London Research Ethics Committee (Ref #0355) and all participants gave written consent of their acceptance to participate in the study.

### Materials

To elicit the false memories, eighteen wordlists were used. Each wordlist consisted of the fifteen most commonly associated words of a critical non-presented word. For example the list *mad, fear, hate, rage, temper, fury, ire, wrath, happy, fight, hatred, mean, calm, emotion, enrage* is composed of the fifteen strongest associates of the word *anger* and whilst the fifteen words in the list were shown to participants, the critical word *anger* was not.

The wordlists were constructed using the first fifteen words listed in the Russell & Jenkins, (1954) norms for the critical non-presented words (see Roediger & McDermott, 1995; Stadler *et al.*, 1999 for full details of list construction). The eighteen wordlists were chosen for their known ability to elicit a high proportion of false memories during recall ( Stadler *et al.*, 1999). The eighteen critical non-presented words used (and their corresponding fifteen wordlists) were: *window, sleep, smell, doctor, sweet, chair, smoke, rough, needle, anger, trash, soft, city, cup, cold, mountain, slow, river* ( Stadler *et al.*, 1999).

The wordlists were put into an automated computerised visual presentation (Microsoft Powerpoint 2007, version 12.0.6654.5000) in which each word was displayed in bold, black ‘Calibri Headings’ typeface, font size eighteen. Each word was displayed in the centre of a white screen at a rate of one second per word, with an inter-word interval of approximately five hundred milliseconds. To mark the start and end of a wordlist a white screen containing a black cross was displayed for one second. Following the end of each wordlist a blank white screen was displayed for two minutes. This coincided with the two minute free recall period (see below). The list order was randomised and the words within each list were presented in order of their associative strength to the critical non-presented word, strongest to weakest.

The recognition test was comprised of one hundred and eight words randomly ordered in four columns of twenty-seven on a sheet of paper. The one hundred and eight words were those from serial positions one, eight, and ten of each of the eighteen studied lists, the eighteen critical lures, and thirty-six unrelated words not found in any of the eighteen lists. The thirty-six unrelated words were selected from the other eighteen word lists published in Stadler *et al.*, (1999) and from the Oxford English Dictionary. The 36 'incorrect' words were: *young, chess, circus, march, ink, rye, keys, chequered, soccer, basket, noon, muscle, piano, scribble, bounce, button, feelers, jail, jubilee, rubric, folder, paint, postcard, fan, lamp, book, computer, first, thought, tile, hide, worth, planet, radio, arm, basement*.

The categorisation test consisted of forty-five printed questions. Each question consisted of five words, three of which were associated with one another and two of which were not. Participants were required to circle the two words that were not associated. An example of a question is as follows: *1. curve, arc, crouch, bend, medicine*, where *curve*, *arc* and *bend* are the three words associated with one another and *crouch* and *medicine* are the words to be correctly circled. Source materials for the categorisation test were example verbal reasoning questions for UK 11+ exams (secondary school entry exams). Questions were reproduced with copyright permission from Coordination Group Publications Ltd ( Parsons, 2002a; Parsons, 2002b), Chukra Ltd, (2007) and Eleven Plus Exam Group, (2010).

### Protocol

All participants were tested in one sitting. Participants were advised that they would be tested on their memory for lists of words and that they would be required to solve some word puzzles.

Participants viewed the visual presentation containing the eighteen wordlists on a large screen (240cm width, 180cm height). At the end of each list a two minute recall period was given. During these free recall periods, participants were instructed to write down as many of the words from the list they had just seen as they could remember. Participants were instructed not to guess, but to only write down words that they were reasonably sure they had seen. Participants were provided with a booklet in which to write down their responses.

Participants then undertook the recognition test. They were instructed to carefully read the words on the sheet provided and to circle any words that they remembered being presented in the eighteen wordlists. Again participants were instructed not to guess but to only circle words they were reasonably sure they had seen.

After the final recall period a ten minute break was given, but participants were instructed not to talk to each other about the study. Participants were then given seven minutes to work through the categorisation test. Again they were instructed not to guess, but to only answer those questions to whose answer they were reasonably sure of. Upon completion participants were fully de-briefed as to the purpose of the study.

### Data analysis

The number of critical non-presented words recalled (false memories), the number of critical non-presented words recognised (false memories), and the number of errors made on the categorisation test were calculated for each individual. These were also converted to give percentage errors (out of those possible to produce) to display graphically. We tested the data for normality and found that the distribution for both the categorisation test and the recognition false memory test departed significantly from a normal distribution (Shapiro-Wilk normality test). Therefore a non-parametric correlation analysis (Spearman’s rank correlation coefficient) was used to look for a potential link between categorisation ability (categorisation test errors) and false memory susceptibility (recall and recognition errors). Additional correlations were used on subsets of the data to check for any biasing effects of priming, outliers and age. Finally, the numbers of recall, recognition and categorisation errors were compared between males and females using Wilcoxon rank sum tests to look for an effect of gender. All analyses were carried out using R statistical software (v.2.14.1). P values below 0.05 were deemed significant.

## Results

False memory susceptibility and categorisation ability Individuals’ susceptibilities to false memories elicited using the Deese-Roediger-McDermott (DRM) paradigm (given as the number of critical non-presented words recalled (Recall False memories, out of a total of 18) and recognised (Recognition False Memories, again out of 18)), and their categorisation abilities (given as the number of questions answered incorrectly on the categorisation test (Categorisation Test Errors, out of a total of 45)).

**Figure 1.**
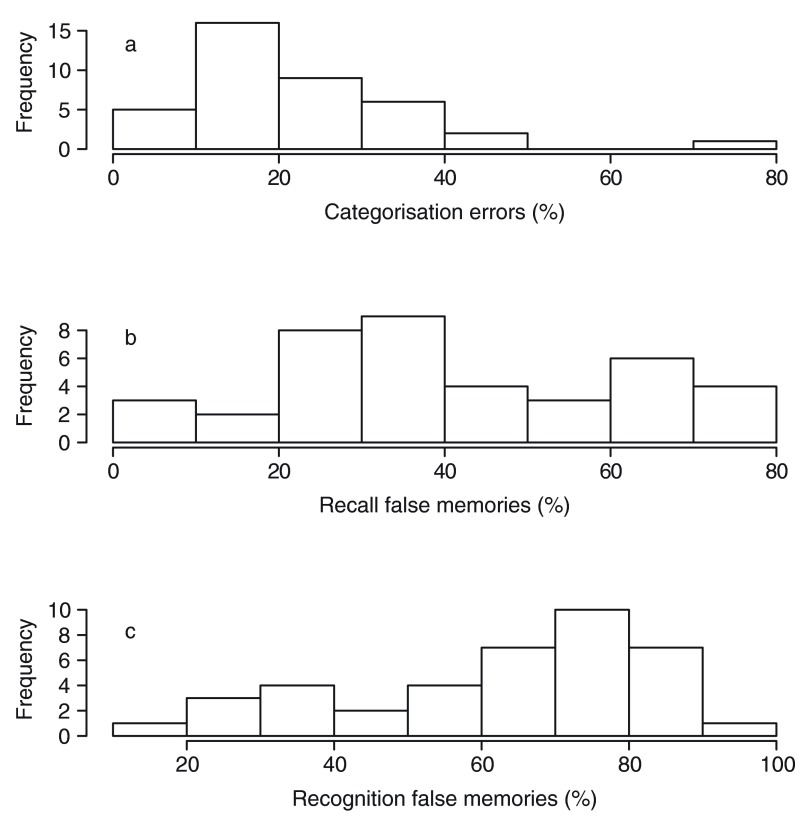
Frequency histograms of individual variation in categorisation ability and false memory performance. **a**) the percentage of errors scored by individuals on the categorisation test, **b**) the percentage of false memories (out of those possible to elicit) recalled by individuals during the DRM paradigm and **c**) the percentage of false memories (out of those possible to elicit) recognised by individuals during the DRM paradigm. N=39. All show a spread of inter-individual variation.

There were substantial inter-individual differences in both participants’ verbal categorisation abilities and their scores on a standardised false memory test. Categorisation errors ranged from 7% to 78% in different individuals (Mean 23%, SD 14%), showing that even though the test we had chosen was originally designed for pre-teens, the task was sufficiently challenging for the tested population to capture a large range of inter-individual variation ( Figure 1a). It was important to establish this since if all participants had near-perfect scores (or indeed if all had equally poor scores), the test would not have been suitable to correlate individual variation with other assessments of cognitive performance.

Variation in individual false memory scores was likewise extensive. Recall false memory scores ranged from 0% to 78% (Mean 41%, SD 21%) of possible false memories made ( Figure 1b). Two individuals did not recall a single critical non-presented word and thus had a score of zero (and 0%) for recall false memories. Conversely three individuals recalled thirteen out of the possible eighteen false memories (and thus scored 72%), and one participant even scored fourteen (78%). Recognition false memory scores ranged from 17% to 94% (Mean 63%, SD 21%) of possible false memories made ( Figure 1c). Five individuals recognised five or less of the critical non-presented words (and thus scored 28% or less), whilst eighteen individuals recognised thirteen or more out of the eighteen possible false memories (and thus scored 72% or more).

**Figure 2.**
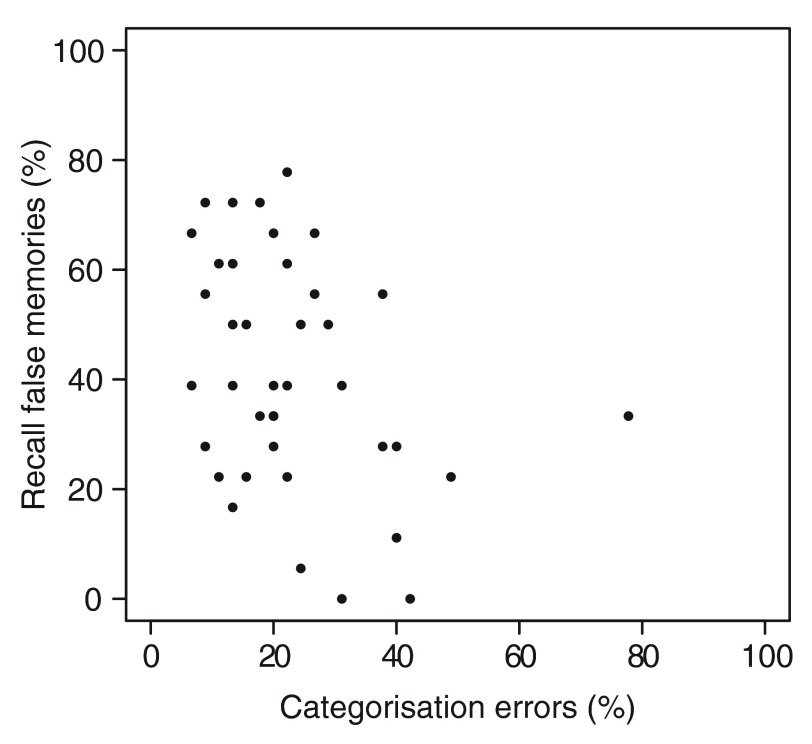
Categorisation ability versus false recall. Individuals’ categorisation abilities (given as the percentage of questions answered incorrectly on the categorisation test) plotted against their susceptibilities to false memories (given as the percentage of critical non-presented words recalled, out of those possible). Those individuals scoring fewer errors on the categorisation test were more susceptible to false memory intrusions and correspondingly had a higher false memory score (r _s_=-0.345, df=37, p=0.032).

We found a significant negative correlation between individuals’ categorisation error scores (given as the number of questions answered incorrectly on the categorisation test) and their false memory susceptibility during free recall (given as the number of critical non-presented words recalled) (r _s_=-0.345, df=37, p=0.032, Figure 2), thus those individuals scoring fewer errors on the categorisation test were more susceptible to false memory intrusions during free recall. In other words, participants that performed worse on the one test performed better on the other, and vice versa – indicating an inter-individual trade-off between categorisation ability on the one hand and false memory susceptibility during free recall on the other.

**Figure 3.**
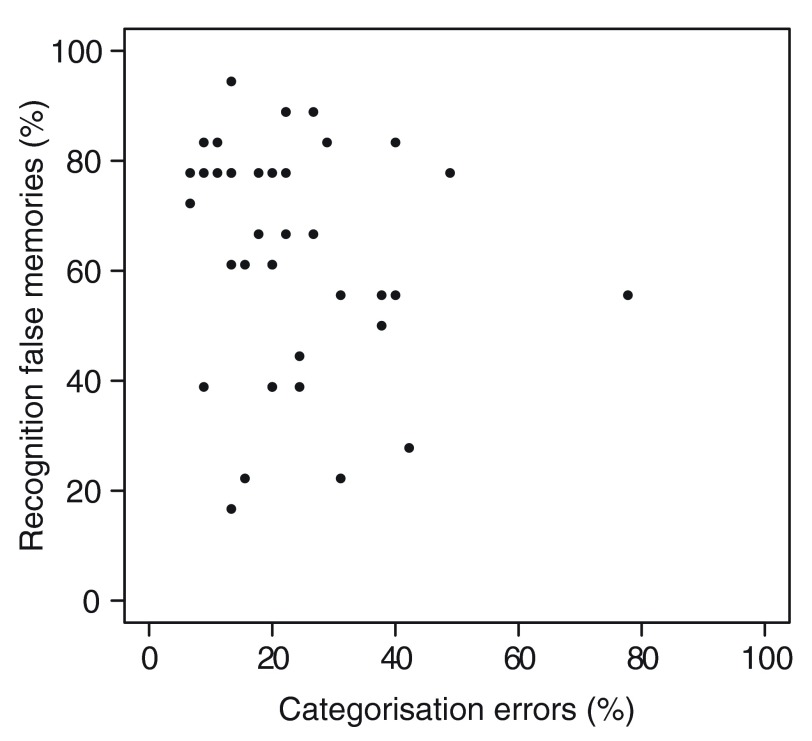
Categorisation ability versus false recognition. Individuals’ categorisation abilities (given as the percentage of questions answered incorrectly on the categorisation test) plotted against their susceptibilities to false memories (given as the percentage of critical non-presented words recognised, out of those possible). Again, those individuals scoring fewer errors on the categorisation test were more susceptible to false memory intrusions and correspondingly had a higher false memory score, though in this case the correlation was not significant: r _s_=-0.202, df=37, p=0.219.

We also found a slight negative correlation between individuals’ categorisation error scores (given as the number of questions answered incorrectly on the categorisation test) and their false memory susceptibility during recognition (given as the number of critical non-presented words recognised); however this trend was not significant (r _s_=-0.202, df=37, p=0.219, Figure 3).

To exclude the possibility that any correlation could be caused by priming, the data were also analysed excluding those categorisation test questions that contained words previously presented in the wordlists, and non-presented as one of the critical non-presented words. In our experiment for example, priming may have meant that the word *eye* presented as part of a question in the categorisation test: *41. Eye neck nose mouth shoulder*, may have been preferentially selected as an answer due to its previous presentation in the word list associated with the critical non-presented word *needle* – *thread, pin, **eye**, sewing, sharp, point, prick, thimble, haystack, thorn, hurt, injection, syringe, cloth, knitting*. As such the scores for twelve questions were removed. A significant negative correlation was still found for free recall and a moderate negative, though non-significant, correlation found for recognition; thus priming cannot account for the result (recall: r _s_=-0.362, df=37, p=0.024, recognition: r _s_=-0.206, df=37, p=0.208).

Additionally, the removal of an outlier (a residuals vs. leverage plot showed a Cook’s distance greater than 0.5 for participant 24, see Dataset 1) did not change the statistical significance of the original result, thus it was not skewing the data unnecessarily in one direction and was therefore not the cause of the significant negative correlation found (recall: r _s_=-0.341, df=36, p=0.036, recognition: r _s_=-0.175, df=36, p=0.293).

The ages of the participants were not greatly varied, with thirty-six out of thirty-nine participants aged nineteen to twenty-one, one participant aged twenty-three, one participant aged thirty and one participant not stating their age. The removal of the data for the participant aged thirty did not change the statistical significance of the original result, thus the greater age of this participant in comparison to the others was also not the cause of the significant negative correlation found (recall: r _s_=-0.387, df=36, p=0.016, recognition: r _s_=-0.251, df=36, p=0.129). Furthermore, the imbalance in the number of male and female participants (seven male, thirty-two female) is unlikely to have caused any bias in the data as there was no significant difference between the two genders in the mean values for the recall errors (Wilcoxon rank sum test: W=114, p=0.956), recognition errors (Wilcoxon rank sum test: W=97.5, p=0.605) nor the categorisation test scores (Wilcoxon rank sum test: W=102, p=0.727).

## Discussion

Our findings show a trade-off between word categorisation ability and semantic false memory susceptibility, so that individuals that make more errors on the false memory test make fewer errors on the categorisation test, and vice versa. Thus our results cannot simply be explained by differences in level of education, literacy, vocabulary or intelligence. If such an underlying factor would have explained performance on *both* tasks, then superior performance on one task would have been a predictor of superior performance on the other task. For example, short term memorisation of word lists recruits working memory, which is often regarded as a general predictor of intelligence ( Oberauer *et al.*, 2005; Oberauer *et al.*, 2008) and likewise the categorisation tests used here are typical components of standardised intelligence tests ( Wechsler, 2004; Wechsler, 2008). Thus one might have predicted a *positive* correlation of error scores in both tasks if an underlying single factor such as intelligence would explain the data. However, the correlation of error scores in the two measured tasks was *negative*. Thus even though this study is clearly correlative in nature, and therefore does not allow us to conclude with certainty that the two performances are based on the same underlying mechanisms, it is intriguing that having a lower tendency to generate false memories comes at a cost, i.e. lower categorisation scores.

To date the majority of scholars interested in false memories have focused on factors which may exacerbate or reduce the occurrence of such memory errors ( Dodson *et al.*, 2000). The adaptive nature of the human memory system as a potential reason for the occurrence false memories has been suggested ( Schacter, 1999; Schacter, 2001), yet the ultimate reasons for their existence has been infrequently explored empirically. More recently, however, evidence has grown for links between individuals’ differing susceptibilities to false memories and their variations in a range of cognitive features. False recall and/or recognition rates in a DRM paradigm have been shown to vary with individuals’ variations in levels of vivid mental imagery ( Winograd *et al.*, 1998), specific area expertise ( Baird, 2003; Castel *et al.*, 2007), working memory capacity ( Watson *et al.*, 2005) and need for cognition (the degree to which an individual actively engages in cognitive tasks) ( Graham, 2007).

Additionally it has been shown that when survival-related (i.e. evolutionarily relevant) information is used in a list-learning paradigm, increased susceptibility to false memories occurs. Howe & Derbish, (2010) found that when participants are asked to process words for their survival value and when the words presented were themselves survival relevant (i.e., *‘death: burial, casket, cemetery, funeral, grave, life, murder, suicide, tragedy, widow*), veridical and false recognition were significantly higher (leading to an overall decrease in net accuracy) than when the words viewed were neutral or negative and were processed for pleasantness. They concluded that whilst it does not at first seem adaptive for survival-related memories to be less accurate and in fact be more prone to false intrusions than other types of memory, it does make sense if considered as a by-product of the adaptive processing of information related to survival. Howe & Derbish, (2010) argue that during the processing of information related to survival, any related information in memory is then primed, which may or may not be *false*, but that this information is then used to guide attention to other survival-related items, which may be crucial in the current situation ( Howe & Derbish, 2010).

It has even been postulated that this greater inaccuracy may actually have adaptive significance, being more helpful in real-world scenarios. For example, in responses to predation threat, false alarms, such as generalising to a large set of cues that might indicate predator presence are clearly less detrimental errors than missing predator presence based on interpreting predators’ cues too narrowly ( Howe & Derbish, 2010). Thus our finding of a significant positive correlation between susceptibility to semantic false memories in a free recall DRM paradigm and word-based categorisation ability, with the creation of these errors a by-product of our ability to group words, is in keeping with recent explorations of the adaptive conditions related to the phenomenon of false memories.

Whilst the age range of the subjects tested was narrow (nineteen to twenty-one years old in the majority) many of the key studies using the DRM paradigm have used only participants also of average undergraduate college study age ( Roediger & McDermott, 1995; Stadler *et al.*, 1999). Additionally the only significant difference in spontaneous false memory creation, caused by the DRM paradigm that is known to occur between participants of different ages, is between children and adults. Several studies have shown that children are less prone to these memory errors, with an increase in their propensity occurring during both childhood and early adolescence ( Brainerd *et al.*, 2002; Brainerd *et al.*, 2004; Forrest, 2002). As such, inferences made from our findings are not just applicable to young adults but should also be pertinent to the ‘average’ adult population as a whole.

Our result of a significant negative correlation between individuals’ errors on a categorisation test and their susceptibilities to semantic type false memories during free recall demonstrates that false memories, to some extent, might be a by-product of our ability to learn rules, categories and concepts. For example, once we have learnt the concept/category of *mammals*, we can identify new animals as members of this category even if we have never seen them before. In this case, labelling the new animal as mammal is not based on false classification, but a correct one based on category membership: the simple flipside of the DRM paradigm, where inferences based on concepts and categories are classed as errors. Thus, our findings add to the increasing body of literature that proposes that some types of false memories might be an inevitable by-product of adaptive cognitive processes as is the case with other memory aberrations ( Abbott & Sherratt, 2011; Beck & Forstmeier, 2007; Newman & Lindsay, 2009).

## Data availability

*F1000Research*: Dataset 1. False memory susceptibility and categorisation ability, http://dx.doi.org/10.5256/f1000research.4645.d31516 ( Hunt & Chittka, 2014).
