# Supplementary material for: False memory susceptibility is correlated with categorisation ability in humans
Source: F1000Res. 2014 Oct 16;3:154. Originally published 2014 Jul 4. [Version 2] doi: 10.12688/f1000research.4645.2 (PMC4168836; doi:10.12688/f1000research.4645.2)
Supplement: Peer review history

# Peer review: Referee response for version 1

**DOI:** 10.5256/f1000research.4966.r5357

**Published:** 2014-09-16

**Authors:** Tom Smulders

**Affiliations:** 1 Centre for Behaviour and Evolution, University of Newcastle, Newcastle upon Tyne, UK

In this paper, Hunt and Chittka show that people who are better at a categorization task also tend to produce more "memory errors" on the DRM paradigm, in which 15 words are shown which are strongly associated with a non-presented word (which is then often (wrongly) recalled or recognized later by the participants). The experiment has been conducted properly (the issue of counterbalancing has been addressed by the priming re-analysis) and the results are clear, although the effect size does not seem large.

The conclusions the authors draw from this result seem eminently reasonable, although they may go a bit beyond the immediate data. After all, they specifically picked a form of false-memory testing that relies heavily on the fact that words in a semantic category are tightly associated with each other. It is then not a great surprise that people who are good at keeping words from the same semantic categories together also show more memory errors. It might have been interesting to ask people to categorize on different criteria than semantic (e.g. based on the letters they contain). Even though this is still a test of finding odd words out in a group, it does not call on semantic categories...

The introduction is probably setting up a bit of a straw man. I think false memories as discussed here should be limited to those in episodic memory. Even the DRM is an episodic memory task (recall the unique list you've just been presented with), which is influenced by semantic associations between words. Therefore, any discussion of selective pressures on memory accuracy should be based on episodic memory alone. And there are lots of debates about what episodic memory is for, and indeed whether accuracy is the most important part of episodic memories. False memories in episodic memories often come about by "intrusion" of more common events into a unique episode. If the common events are that common, the unique exception may not be important to remember and indeed may interfere with the (adaptive) application of a learned rule. So there may be many arguments against the idea that memory should always be accurate. Nevertheless, this does not take away from the data or the final conclusions of this paper.

I have read this submission. I believe that I have an appropriate level of expertise to confirm that it is of an acceptable scientific standard.

# Response

**Published:** 2014-10-17

**Authors:** Lars Chittka

**Affiliations:** School of Biological and Chemical Sciences, Queen Mary, University of London, UK

We thank the referee for the constructive comments. We agree with the referee that one function of cognition (probably not just episodic memory) is to extract commonalities between distinct individual memories, learn rules, and to categorise objects and events by common properties, and to classify novel objects or events into existing frameworks. Certainly such rule-learning and classification abilities are adaptive, and we have thus deleted the statement that “false memories cannot in themselves be useful”.

Nonetheless, it is probably fair to say that many of the contexts in which false memories occur ‘naturally’, as well as the common experimental paradigms (not just ours), rely on rule learning and classification abilities. Witnesses of a nocturnal street robbery might describe the perpetrator as a hooded teenager, when it later turns out that the assailant was middle-aged and balding. While such false memories create enormous difficulties for the police and criminal courts, the witnesses may have made of use of their (typically, but clearly not always, adaptive) ability to learn rules about settings or conditions that, at least potentially, indicate danger. From the earliest explorations of false memories, such as the transmission chain studies in Bartlett’s work, it was investigated how distorted memories fall in with previously memorised rules, cultural norms etc.  Whether this means that individuals that are better at extracting common properties incur a cost when it comes to storing large number of individual items/memories is precisely the subject of this study. We agree with the referee that it might sometimes be more useful to memorise common events than individual ones, or indeed to extract the commonalities between events and discard their idiosyncrasies, and this is hopefully clearer in the revised version.

# Peer review: Referee response for version 1

**DOI:** 10.5256/f1000research.4966.r5492

**Published:** 2014-08-08

**Authors:** Rob Nash

**Affiliations:** 1 Department of Psychology, University of Surrey, Guildford, Surrey, UK

This is an interesting article that has good relevance to ongoing debates on the adaptiveness of memory errors. The methods and analyses are appropriate and the authors have generally represented the scientific literature well. I have a few minor suggestion for improvement, as follows:

In the paragraph beginning “ *While such tests might be viewed as rather remote from real-life situations..*” it should be noted that there are several more studies in which errors in different false memory paradigms are correlated either weakly (e.g., Zhu *et al.*, 2013) or not at all (e.g. Ost *et al*., 2013).

The line “ *Clearly false memories cannot in themselves be useful*” is disputable – several studies now show positive consequences of distorted memories, see e.g. Howe, Garner & Patel, 2013; Bernstein & Loftus, 2009).

Line *“It is therefore possible that memory errors are an inevitable fluke of a powerful, adaptive cognitive phenomenon”* – I would prefer to say *“some memory errors”* – there is a broad literature on other adaptive reasons why memory errors occur, see e.g. Newman & Lindsay (2009). Same point applies to the very final sentence of the Discussion.

Why wasn't the order of the two tasks counterbalanced? Is it plausible that the first (memory) task might have primed a particular mindset in participants that affected their categorization performance? I’d suggest the addition of a little discussion of this point.

Data analysis – which data departed from normality? The authors have reported a normality test but it isn't clear to which variable this test pertains.

The line *“A significant negative correlation was still found for free recall and a moderate negative correlation still found for recognition*” – the authors should reiterate explicitly that the latter correlation was non-significant.

I have read this submission. I believe that I have an appropriate level of expertise to confirm that it is of an acceptable scientific standard.

# Response

**Published:** 2014-10-17

**Authors:** Lars Chittka

**Affiliations:** School of Biological and Chemical Sciences, Queen Mary, University of London, UK

We thank the referee for the constructive comments. We have taken all of them on board in the revised version. We have also added the five additional references indicated by the referee, and modified the text in line with the contents of these publications. We have added the requested information about non-normality of some of the data.

The referee asks why we have not used a counterbalanced design, so that in addition to setting the false memory test first and the categorisation test second, we might have reversed the order. This would indeed be a useful procedure to exclude the possibility that any correlation could be caused by priming, occurring as a result of words occurring in both tests. However, to address the potential complication of priming, we had already reanalysed the data excluding those categorisation test questions that contained words previously presented in the DRM word lists, and non-presented as one of the critical non-presented words. However, even after removal of all individual tasks where such words occurred, the overall results remained unaltered (see results section).
